# Patient-derived xenograft in zebrafish embryos: a new platform for translational research in gastric cancer

**DOI:** 10.1186/s13046-017-0631-0

**Published:** 2017-11-15

**Authors:** Jia-Qi Wu, Jing Zhai, Chong-Yong Li, Ai-Min Tan, Ping Wei, Li-Zong Shen, Ming-Fang He

**Affiliations:** 1 Institute of Translational Medicine, College of Biotechnology and Pharmaceutical Engineering, Nanjing Tech University, 30 Puzhu South Road, Nanjing, 211800 People’s Republic of China; 2 Division of Gastrointestinal Surgery, Department of General Surgery, First Affiliated Hospital, Nanjing Medical University, 300 Guangzhou Road, Nanjing, 210029 People’s Republic of China; 3 Department of Surgery Oncology, First Affiliated Hospital, Nanjing University of Traditional Chinese Medicine, Nanjing, Jiangsu 210029 China; 4 State Key Laboratory of Translational Medicine and Innovative Drug Development, Jiangsu Simcere Pharmaceutical R&D Co. Ltd., Nanjing, Jiangsu 210042 China

**Keywords:** Gastric cancer, Zebrafish, Patient-derived xenograft, Microinjection, Translational research

## Abstract

**Background:**

Gastric cancer (GC) is among the most commonly cancer occurred in Asian, especially in China. With its high heterogeneity and few of validated drug targets, GC remains to be one of the most under explored areas of precision medicine. In this study, we aimed to establish an in vivo patient-derived xenograft (PDX) model based on zebrafish (*Danio rerio*) embryos, allowing for a rapid analysis of the angiogenic and invasive potentials, as well as a fast drug sensitivity testing.

**Methods:**

Two human gastric cancer cell lines (AGS and SGC-7901) were xenografted into zebrafish embryos, their sensitivity to 5-FU were tested both in vitro and in vivo. Fourteen human primary cells from gastric cancer tissue were xenografted into zebrafish embryos, their proliferating, angiogenic and metastatic activities were evaluated in vivo. Sensitivity to 5-FU, docetaxel, and apatinib were also tested on primary samples from four patients.

**Results:**

SGC-7901 showed higher sensitivity to 5-FU than AGS both in vitro (6.3 ± 0.9 μM vs.10.5 ± 1.8 μM) and in vivo. Nine out of fourteen patient samples were successfully transplanted in zebrafish embryos and all showed proliferating, angiogenic and metastatic potentials in the living embryos. Four cases showed varied sensitivity to the selected three chemotherapeutic drugs.

**Conclusions:**

Our zebrafish PDX (zPDX) model is a preclinically reliable in vivo model for GC. The zPDX model is also a promising platform for the translational research and personalized treatment on GC.

**Electronic supplementary material:**

The online version of this article (10.1186/s13046-017-0631-0) contains supplementary material, which is available to authorized users.

## Background

Gastric cancer (GC) remains a major health burden across the globe. According to the 2012 statistics of the International Agency for Research on Cancer (IARC), GC was the fifth most common malignancy and the third leading cause of cancer death worldwide [1]. More than 70% of cases occur in developing countries, and half the world total occurs in Eastern Asia (mainly in China). According to the statistics of Chinese National Office for Cancer Prevention and Control, GC was the 2nd and 3rd commonly diagnosed cancers among men and women respectively, and was the 2nd leading causes of cancer death among both sexes [2].

In China, nearly 90% of patients were diagnosed as advanced gastric cancer [3]. Although the addition of targeted drugs has improved the prognosis to some extent in recent years, the comprehensive treatment based on fluorouracil containing chemotherapy remains the main strategy for advanced GC. The median survival rate of patients with advanced GC is still less than 12 months, and the overall 5-year survival rate of GC is as low as only 20%, these are due to the chemoinsensitivity or chemoresistance of GC resulting from the high heterogeneous nature and other unknown mechanisms [4]. Therefore, development of new drugs, as well as establishment of personalized treatment strategies for current drugs becomes the principal challenges.

Patient derived xenograft (PDX) model have become popular in recent years with more advantages than cell line-derived xenograft (CDX) model. It closely recapitulates the heterogeneity of patients’ primary tumors and possesses biological stability of gene-expression and mutational status. Increasing amounts of evidence have suggested that PDX model faithfully recapitulate patient tumor biology and predict patient drug response by directly comparing drug responses in patients and their corresponding xenografts [5–8]. Nowadays, most PDX models are established by subcutaneously transplanting tumor tissues of patients into NOD/SCID (non-obese diabetic/severe combined immunodeficiency) mice. Mice PDX models from various tumors have been established, including colorectal cancer [9], breast cancer [10], non-small cell lung carcinoma [11], renal cell carcinoma [12] and gastric cancer [13] for the study of tumor biology and drug screening. However, the associated cost is substantial and the time it takes to complete these studies can be extensive and not compatible with patient-directed interventions in an actionable time frame.

The teleost zebrafish (*Danio rerio*) is a powerful and genetically tractable model to study human malignancies. It shows high levels of physiologic and genetic similarities to mammals, closely mimicking the clinical setting and permitting the natural history of the tumor to be monitored [14]. The transparent embryos display distinct features that facilitate the exploration of tumor development, angiogenesis, invasion and metastasis, which makes it a promising xenograft tumor model [15, 16]. In recent years, the value of zebrafish PDX (zPDX) model has just been emerged [17–19].

The aim of this paper is to describe a new in vivo zPDX model of GC. This model can be used to study tumor angiogenesis, cell invasiveness and drug responses in a time-saving and cost-saving manner. Importantly, this work shed light on the zPDX model to serve as the first real-time in vivo platform for personalizing GC treatment.

## Methods

### Reagents

5-Fluorouracil (5-FU, purity ≥98%) were purchased from Sigma Aldrich (St. Louris, MO, USA) and was dissolved in embryo medium or cell culture medium to obtain a stock concentration of 5 M. Docetaxel (purity ≥98%) and apatinib (purity ≥98%) were purchased from MERYER (Shanghai, China) and were dissolved in DMSO to obtain stock concentrations of 5 mM and 500 μM respectively. Fetal bovine serum (FBS), phosphate buffer saline (PBS), Roswell Park Memorial Institute basal medium 1640 (RPMI 1640), penicillin and streptomycin were purchased from Basal Media Technologies (Shanghai, China).

### Zebrafish care and handing

Transgenic zebrafish *Tg(fli-1:EGFP)* expressing enhanced green fluorescent protein (EGFP) in the endothelial cells were obtained from Model Animal Research Center of Nanjing University. They were kept at 28.5 °C as described [20]. The light-dark cycle was 14:10 h. Embryos were obtained by mixing 2 males and 2 females in tanks equipped with a grid to avoid the predation of newly spawned eggs. Fish were mated and spawning was stimulated by the onset of light. Embryos were collected and placed at 28.5 °C in Petri dishes containing embryo medium (0.2 g/L of Instant Ocean® Salt in distilled water). The whole embryos spawned were pooled, counted and the malformed embryos were discarded. Spawns demonstrating low fertilization rate (< 85%) or frequent developmental abnormalities (> 5%) were not used. Embryos were staged according to Kimmel et al. [21]. The age of the embryos is indicated as hours post fertilization (hpf). The zebrafish studies were approved by the Institutional Animal Care and Use Committee (IACUC) at Nanjing Tech University.

### Cell line culture and primary tissue dissociation

The GC cell lines AGS and SGC-7901 (ATCC, Rockville, MD, USA) were cultured in RPMI 1640 supplemented with 10% FBS and 100 U/mL penicillin and streptomycin. Gastric cancer samples, from September 2016 to August 2017, were obtained from the Division of Gastrointestinal Surgery, Department of General Surgery, First Affiliated Hospital, Nanjing Medical University after patients’ informed consent and Institutional Ethics Committee approval (number of registry 10,092). The research containing human subjects was carried out according to The Code of Ethics of the World Medical Association (Declaration of Helsinki). All patients did not receive radiation or chemotherapy before surgical resection. The tissue samples were transferred directly into the pre-chilled tissue storage solution (Miltenyi, BergischGladbach, Germany) after resected. Primary single cells from the tissue samples were isolated through the tumor dissociation kit (Miltenyi, BergischGladbach, Germany) following manufacturer’s instructions.

### In vitro cell viability assay

Cell viability was measured using a cell counting kit-8 (CCK-8, Dojindo, Japan). The cells at logarithmic phase were seeded in 96-well plates (3 × 10^3^ cells/well), after overnight incubation, the medium was replaced with the fresh medium (150 μl/well) containing indicated concentrations of 5-FU for 72 h. Cells treated with culture medium served as vehicle control. Subsequently, 10 μl of CCK8 solution was added to each well, and the cells were incubated for 2 h. The absorbance was measured at 450 nm using a microplate reader (BioTek, Winooski, VT, USA). The absorbance in the control group was regarded as 100% cell viability. The results were expressed as the percentage of inhibition in the form of absorbance. The 50% inhibition concentration (IC_50_) was determined by GraphPad Prism 5.0. All experiments were done in triplicate and independent experiment was repeated at least four times.

### Cell labeling, xenograft and enumeration procedure

Cell lines AGS and SGC-7901 and primary GC cells were fluorescently labeled with CM-DiI (Invitrogen, Life Technologies, Carlsbad, CA, USA) according to the manufacturer’s instructions. Labeled cells were washed in PBS twice, re-suspended in RPMI1640 supplemented with 10% FBS at 2 × 10^7^ cells/ml. Cell viability was assessed by trypan blue staining before the injection. Cell viability was higher than 95% for GC cell lines and 70% for primary GC cells.

**Fig. 1:**
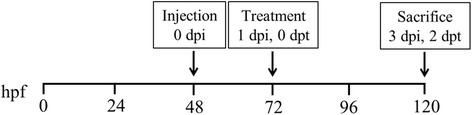
Schematic of in vivo zebrafish xenograft and drug treatment. Hpf: hours post fertilization, dpi: days post injection, dpt: days post treatment

Transgenic zebrafish embryos *Tg(fli-1: EGFP)* at 24 hpf were dechorionated with 1 mg/ml of pronase (Sigma-Aldrich, St. Louris, MO, USA). After removing the chorion, embryos were soaked in embryo medium with 0.2 mM 1-phenyl 2-thiourea (PTU) and incubated for further 24 h at 28.5 °C. At 48 hpf, the embryos were anesthetized with 0.0003% tricaine (Sigma-Aldrich, St. Louris, MO, USA) and positioned with their right side up on a wet agarose pad. Approximately 200–300 cells for cell line and approximately 600–800 primary cells for patient samples [17, 18, 22–24] were injected into yolk sac per zebrafish embryo using a microinjector (IM-31, Narishige, Japan) while under the observation by stereoscope (SMZ 745 T, Nikon, Japan). After transplantation, embryos were incubated for 1 h at 28.5 °C, checked for presence of cells at yolk sac and the absence of cells in the circulating system. Then embryos were incubated at 32 °C for the following days (Additional file 1: *Materials and Methods* and Additional file 2: Figure S1 and Additional file 3: Figure S2). A group of 10 embryos was sacrificed and dissociated into a single cell suspension, the number of CM-DiI-labeled cells was enumerated to be the base-line number of GC cells prior to treatment with vehicle or drug to ensure cells engraft and proliferate in the zebrafish embryos [25]. To confirm the enumeration of human cancer cells at indicated time points and exclude the non-specific staining of tissue debris, co-staining with 10 nM DRAQ5 (Biostatus Ltd. Leicester, UK) nuclear stain was used (Additional file 4: Figure S3). A schematic chart indicated the time line for cell injection and drug treatment (Fig. 1).

### Drug administration by soaking and microinjection to the xenograft model

For drug delivery by soaking, drug exposure by addition to the larval water, xenografted zebrafish embryos at 72 hpf were transferred randomly to 24-well plates, 10 embryos per well with 0.5 ml of embryo medium containing various concentrations of drugs (docetaxel and apatinib) for a treatment period of 48 h. Zebrafish embryos treated with 0.1% DMSO was used as a vehicle control. Treatment experiments were carried out at a constant temperature (32 °C) in the dark. For drug delivery by microinjection, drug (5-FU) were diluted to proper concentrations by embryo medium for yolk sac microinjection. Before microinjection, zebrafish embryos at 72 hpf were anesthetized with 0.0003% tricaine (Sigma-Aldrich, St. Louris, MO, USA) and positioned with their right side up on a wet agarose pad. 10 nl of the drug at designated concentrations was injected into yolk sac per zebrafish embryo using a microinjector (IM-31, Narishige, Japan) while under observation by stereoscope (SMZ 745 T, Nikon, Japan). Injected embryos were transferred randomly to 24-well plate, 10 embryos per well with 0.5 ml of embryo medium for a treatment period of 48 h. Zebrafish embryos injected with 10 nl embryo medium served as a vehicle control. A schematic chart indicated the time line for cell injection and drug treatment (Fig. 1). For angiogenesis observation, xenografted embryos were treated with 50 nM VRI by soaking at 6 hpi and incubate at 32 °C. Photos were taken at 72 hpf.

### Imaging

We monitored tumor cell growth and migration in vivo at 1, 4 and 7 days post injection (dpi) by an inverted fluorescence microscope (IX71, Olympus, Japan). Angiogenesis was observed by confocal microscope (LSM710, ZEISS, Germany). We considered as active migration if the labeled gastric cells were identified outside the yolk sac region (in the head, trunk and/or tail) [19].

### Immunohistochemical methods

Embryos with injected cells at (7 dpi) were fixed in 4% paraformaldehyde, dehydrated, paraffin embedded, and sectioned (6–8 μm). Sections were stained with hematoxylin and eosin. The images were acquired with inverted fluorescence microscope (IX71, Olympus, Japan).

### Statistics

All statistical analyses were expressed as mean ± SEM using GraphPad Prism 5.0. The decrease/increase in fold of change was analyzed using one-way ANOVA followed by Dunnett multiple comparison test. Significance was considered when *P* values were lower than 0.05. (***) indicates statistical significance *P* < 0.005, (**) *P* < 0.01, (*) *P* < 0.05. All experiments were done in triplicates and independent experiment was repeated at least three times.

## Results

### Cell lines AGS and SGC-7901 induced angiogenesis in zebrafish embryos

**Fig. 2:**
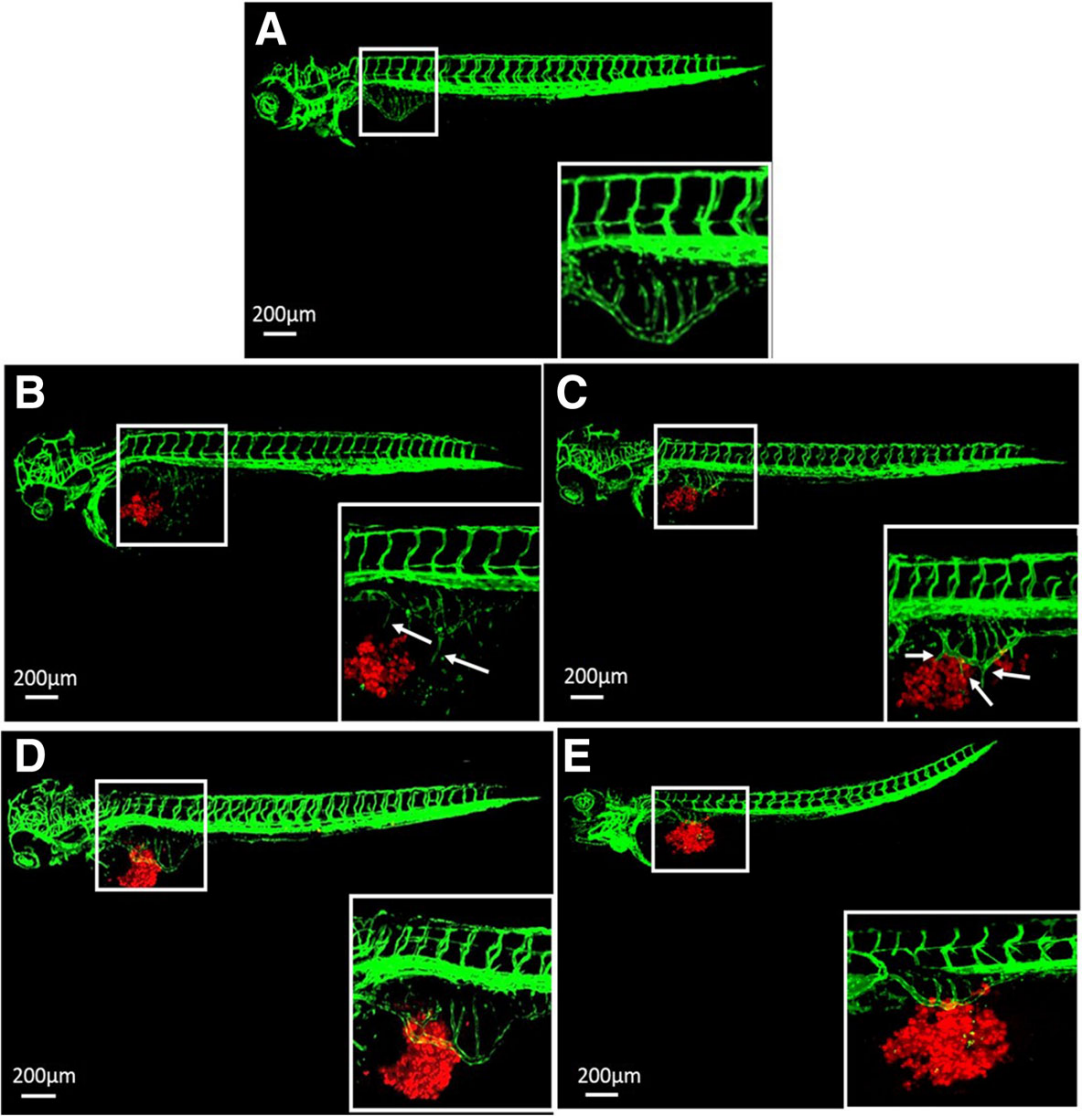
Gastric cancer cells survived and induced angiogenesis in larval zebrafish (*fli-eGFP*). **a** Typical images of subintestinal vessels of uninjected embryo at 3 dpf. AGS cells (**b**) and SGC-7901 cells (**c**) were injected to the zebrafish embryos, and induced angiogenesis at 1 dpi. 50 nM VRI can block angiogenesis of the subintestinal vessels caused by cell lines AGS (**d**) and SGC-7901 (**e**). The white boxes at lower right corner showed the higher magnification of the upper left white boxes. The arrow indicated the tumor cell-induced angiogenesis. Hpf: hours post fertilization; dpi: days post injection

During the time frame from 2 dpf to 3 dpf, the intact subintestinal vessels (SIVs) of zebrafish embryos form and look like a basket (Fig. 2a). The injected cell lines AGS (Fig. 2b) and SGC-7901 (Fig. 2c) showed pro-angiogenic behaviors in zebrafish embryos as early as 1 day post injection (dpi), the SIVs of the embryos formed additional branches and sprouted towards the tumor implantation mass (Fig. 2b, c). VRI, a pyridinyl-anthranilamide compound that displays strongly inhibition of the kinase activities of both VEGFR-1 and 2, could block the angiogenesis induced by both AGS (Fig. 2d) and SGC-7901 (Fig. 2e).

### Cell line SGC-7901 showed higher sensitivity to 5-FU than AGS both in vitro and in vivo

**Fig. 3:**
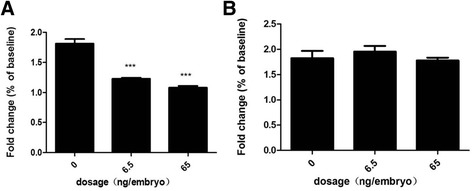
Proliferation of gastric cell lines in the zebrafish xenografted model under different treatment groups of 5-FU at 2 dpt. 200–300 cells of AGS cell line (**a**) and SGC-7901 cell line (**b**) were fluorescently labeled in red and microinjected to the yolk sac of each zebrafish embryo at 48 hpf (0 dpi). After 24 h (0 dpt), zebrafish embryos were microinjected with vehicle control (0.1% DMSO), 6.5 ng/embryo, and 65 ng/embryo of 5-FU respectively for the 2 days. Cell number was determined by sacrificing embryos at 0 dpt and 2 dpt. Cell number at 0 dpt was normalized to 1 and set as baseline. Quantitative values are means ± SEM from 30 independent individuals. Significance at different treatment group was considered when *P* values were lower than 0.05. (***) indicates statistical significance *P* < 0.001. hpf: hours post fertilization, dpi: days post injection, dpt: days post treatment

In the in vitro chemosensitivity assay, 5-FU showed significant inhibition of the cell viability on both cells in a dose-dependent manner (Additional file 5: Figure S4). At a 72 h-incubation time, the IC_50_ were 10.5 ± 1.8 μM and 6.3 ± 0.9 μM for AGS and SGC-7901 respectively (Table 1), in which SGC-7901 showed higher sensitivity to 5-FU treatment compared to AGS in vitro. Our result agreed with Chen’s report that AGS was relatively resistant to 5-FU treatment than SGC-7901 [26]. Next, AGS and SGC-7901 cells were xenografted into zebrafish embryos at 48 hpf separately. At 72 hpf, 50–4000 μM of 5-FU were administrated to the embryos by soaking. To our surprise, none of the concentrations caused inhibition of tumor growth, as well as any adverse effect on the embryo development at 2 days post treatment (dpt, data not shown). We then tried drug delivery by microinjection. The maximum tolerated doses (MTD) of 5-FU was determined as 65 ng/embryo (Additional file 1: *Materials and Methods* and Additional file 6: Figure S5). The cell number at 0 dpt was set as the baseline and was normalized to 1. At 2 dpt, the SGC-7901 cells proliferated by 1.8 folds in the control group, but by 1.2 and 1 folds in the 6.5 and 65 ng/embryo of 5-FU treatment groups respectively (Fig. 3a). AGS cells proliferated by 1.8 folds in the control group at 2 dpt, but by 1.9 and 1.7 folds in the 6.5 and 65 ng/embryo of 5-FU treatment groups respectively (Fig. 3b). SGC-7901 showed higher sensitivity to 5-FU treatment compared to AGS in vivo.

**Table 1:** IC_50_ values of 5-FU on SGC-7901 and AGS

Cell line	IC_50_(μM)
SGC-7901	6.3 ± 0.9
AGS	10.5 ± 1.8

### Patient-derived gastric cancer samples formed adenoid structure, induced angiogenesis and presented metastasis character

**Fig. 4:**
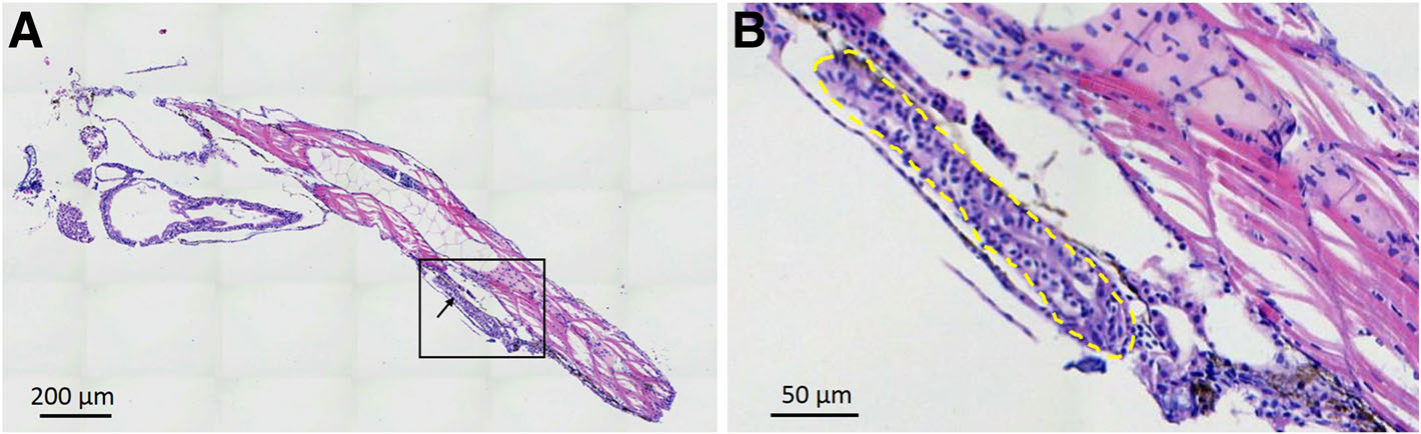
Histological hematoxylin and eosin (H & E) staining of the whole-mount zPDX model at 7 dpi. A low (**a**) and a higher (**b**) magnification of a representative zPDX were showed. Black box in (A) indicates the area of zoom. Arrows in (A) and yellow dashed line in (B) point to the adenoid structure formed by primary epithelial cells from GC patient. Dpi: days post injection

**Fig. 5:**
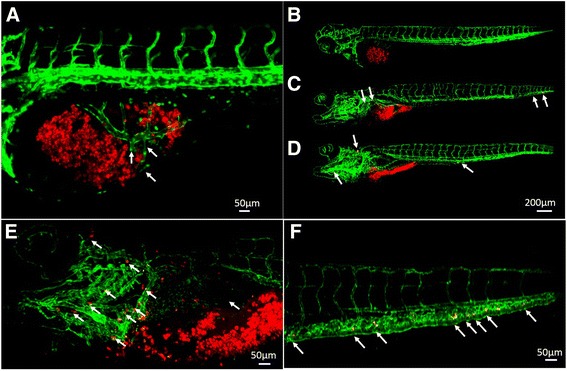
Primary cells from GC tissue induced angiogenesis and metastasized in larval zebrafish (*fli-eGFP*). 600–800 primary cells for patient samples were fluorescently labeled in red and microinjected into yolk sac of each zebrafish embryo at 48 hpf (0 dpi). Primary cells from GC tissue induced angiogenesis at 1 dpi (**a**), and showed invasive behaviors at 1 dpi (**b**), 4 dpi (**c**) and 7 dpi (**d**) in zebrafish PDX model. At 7 dpi, we could detect cancer cells in the brain (**e**), and the caudal hematopoietic tissue (CHT) region (**f**). Arrow in (**a**) points to the tumor induced new blood vessels within tumor mass. Arrows in (**c**)-(**f**) point to the metastasized tumor cells in the head, trunk and tail of the zebrafish embryos. Hpf: hours post fertilization, dpi: days post injection

In the transverse histological sections at the PDX level (7 dpi), primary cells from GC tissue also formed adenoid structure [27] in zPDX models which generally conserved their original feature (Fig.4). Primary cells from GC tissue induced angiogenesis at 1 dpi. Zebrafish vasculature penetrated into the xenografted tumor mass (Fig.5a). Primary cells from GC tissue labeled in red did not invade at 1 dpi (Fig.5b), but showed progressive and extensive dissemination throughout the developing embryo and metastasis toward brain, trunk and tail at 4 dpi (Fig.5c) and 7 dpi (Fig.5d). Brain has a high vasculature density. Tumor cells could be found in the brain at almost single cell resolution (Fig. 5e). Tumor cells could also be found in the caudal hematopoietic tissue (CHT) region (Fig. 5f), which is a “hot spot” region for tumor cell to extravasate from vessels and invade adjacent tissues [24, 28].

### 64% samples of GC patient showed successful transplantation

Primary GC cell xenografts were performed from fourteen patients. Their clinical and histopathological characteristics were summarized in Table 2. Nine out of fourteen primary GC cells were successfully transplanted into zebrafish embryos, whereas in five cases the xenograft was not successful because of the viscous texture which cannot be dissociated to single cells and caused subsequent needle clogging. In the nine cases of successful zebrafish PDX models, all nine primary gastric cancer samples showed cellular active migration in the developing zebrafish embryos.

**Table 2:** Clinical and histopathological characteristics of patients with gastric cancer enrolled to generate PDXs in zebrafish

Patient #	Sex	Age (years)	Tumor size(cm)	Pathological type	Lauren	T N M	Number of metastasis lymph nodes
1	M	64	4.5 × 4 × 1.5	Adenocarcinoma (II-III)	Intestinal	3 2 0	6/42
2	M	66	4 × 3 × 1	Adenocarcinoma (II-III)	Hybrid	2 2 0	5/39
3	M	56	2 × 2 × 1.2	Adenocarcinoma (II-III)	Hybrid	3 0 0	0/53
4	F	47	6.5 × 5 × 1.5	Adenocarcinoma (II-III)	Intestinal	3 0 0	0/39
5	M	64	4 × 3 × 2.5	Poorly differentiated adenocarcinoma	Diffuse	4 3b 0	26/52
6	M	64	4.5 × 2.5 × 1	Adenocarcinoma (II-III)	Hybrid	2 0 0	0/31
7	M	64	4 × 3.3 × 0.5	Adenocarcinoma (II-III)	Hybrid	3 2 0	4/33
8	M	67	7 × 6.3 × 3	Poorly differentiated adenocarcinoma	Diffuse	4 1 0	1/31
9	M	62	10.5 × 9 × 2.1	Adenocarcinoma (II-III)	Intestinal	4 3 0	8/46

*M* male, *F* female

### Preclinical drug sensitivity studies in the zebrafish PDX model

**Fig. 6:**
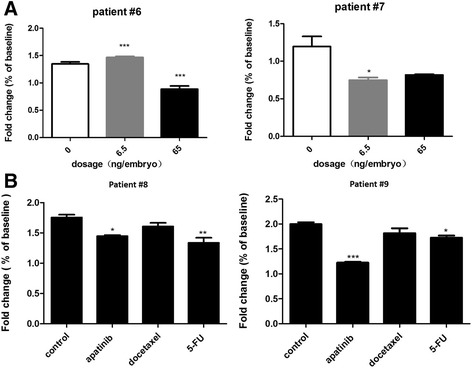
Quantification of patient samples (#6 - #9) engraftment with three chemotherapeutic drugs. Patient samples (#6 and #7) were treated with 5-FU at 6.5 ng/embryo and 65 ng/embryo for 2 days (**a**). Patient samples (#8 and #9) were treated with apatinib (0.5 μM), docetaxel (5 μM), and 5-FU (65 ng/embryo) for 2 days (**b**). Cell number at 0 dpt was normalized to 1 and set as baseline. Fold change was determined by sacrificing embryos at 0 dpt and 2 dpt. Quantitative values are means ± SEM from 30 independent individuals. Significance at different treatment group was considered when *P* values were lower than 0.05. (*) indicates statistical significance *P* < 0.05 and (***) *P* < 0.001. dpt: days post treatment

In clinic, the primary curative treatment of GC is surgical resection [29]. However, 70–80% of these patients with lymph node metastases will relapse and die of their disease [30]. Chemotherapy including 5-FU and docetaxel in postoperative adjuvant treatment has been shown to prolong survival and improve a high quality of life [31, 32]. Apatinib is a novel receptor tyrosine kinase inhibitor which selectively targeting the intracellular ATP-binding site of vascular endothelial growth factor receptor 2 (VEGFR-2). It is an orally-bioavailable agent currently being studied in several solid tumor types and showing a promising activity in gastric cancer [33, 34]. It was approved by SFDA of China for the treatment of advanced gastric cancer in 2014. We next evaluated whether this model could be used to assess efficacy of anti-GC agent, like 5-FU, docetaxel, and apatinib. We selected zebrafish PDX models from four GC patients (patient #6-#9), among which, patient #6 and #7 were diagnosed GC and received curative surgery at January 2017, whereas patient #8 and #9 have just received curative surgery at August 2017. All four patients received postoperative adjuvant treatment of 5-FU. Zebrafish PDX model of patients #6 and #7 were subject to 5-FU treatment at two dosages of 6.5 and 65 ng/embryo. Cells were enumerated at 0 dpt and 2 dpt. Cell number at 0 dpt was set as baseline and normalized to 1. Cells from patient #6 in the control group proliferated by 1.4 folds from 0 dpt to 2 dpt. 5-FU at 6.5 ng/embryo slightly stimulated cell proliferation by 4% whereas inhibited cell proliferation by 40% at dosage of 65 ng/embryo (Fig.6a). Cells from patient #7 in the control group proliferated by 1.2 folds from 0 dpt to 2 dpt. 5-FU treatment caused inhibition of cell proliferation by 38% and 32% at dosage of 6.5 and 65 ng/embryo respectively (Fig.6a). Paitient #7 showed a higher sensitivity to 5-FU treatment compared to patient #6. We next selected patient #8 and #9 to test the response to a wider range of chemotherapeutic drugs, such as docetaxel and apatinib. The MTD of docetaxel and apatinib were determined as 5 μM and 0.5 μM (Additional file 1: *Materials and Methods* and Additional file 6: Figure S5). In Fig. 6b, cells from patient #8 in the control group proliferated by 1.75 folds from 0 dpt to 2 dpt. 5-FU at 65 ng/embryo inhibited cell proliferation by 23%, Apatinib at 0.5 μM by 18% and docetaxel at 5 μM by 8%, respectively. These data suggested that patient #8 is sensitive to 5-FU. Cells from patient #9 in the control group proliferated by 2 folds from 0 dpt to 2 dpt. Apatinib at 0.5 μM caused inhibition of cell proliferation by 39%, 5-FU at 65 ng/embryo by 14% and docetaxel at 5 μM by 9%, respectively. These data suggested that patient #9 is sensitive to apatinib. Patient #7 showed a good response to 5-FU treatment in our zebrafish PDX model. He also showed no indication of relapse 8 month after surgery. There is a good correlation between our zebrafish PDX model and clinic result. But unfortunately, we lost contact with the patient #7, thus we could not get the clinic information about relapse or no relapse. Patient #8 and #9 have just received curative surgery of 5-FU, their clinic information about relapse or not will be tracked.

## Discussion

Here we described a zPDX model of GC, which offers several advantages over other animals, like mouse. First, the short generation time, the large number of offsprings, the transparency of the embryos which enables noninvasive imaging, the external development and the small size of the embryos make zebrafish a more practical and less expensive laboratory system [19]. Second, the transparency of the embryos could facilitate the visualization of tumor cell behaviors and its interactions with the microenvironment such as host blood vessels [22, 35]. Third, the ability of using less patient cell numbers (200–800 cells/embryo vs. 1 million cells/mouse) [36], the efficiency of zPDX model for drug screening right after cell dissociation (cell at passage 0 in zebrafish vs. passage 3 or after in mouse) that preserves a better human origin [13, 18, 37], the power of performing medium throughput in vivo drug screening with a short latency (7 days in zebrafish model vs. several weeks to months in mouse model) that enable quick screening in real time [13, 18]. However, for the purpose of in vivo passage and amplification of human tumor samples to establish cryopreserved tissue bank like mouse PDX (mPDX) model, adult zebrafish could provide an alternative to the zebrafish embryo [38].

In our current research, we collected 14 patient samples of GC, out of which, 9 were successfully established the zPDX model. The success rate of transplantation was 64%, lower than of the reported (75%) on neuroendocrine tumor zPDX model [19], but much higher than that of the reported (34%) on mPDX model of GC [13]. Due to the heterogeneity of the cancer cells, currently, we barely know what cell population in the collected samples, which might be the reason why the success of transplantation varies among different samples. Meanwhile, the technical issue might be another reason of transplantation failure [19]. In our experiment, at 1 h after cell microinjection, all dead embryos, or without uniform cell graft and with cells in the circulation system, were discarded, giving the success rates of microinjection between 40 and 70%, similar to that of reported by Marques’s study [24, 37]. Practically, 600–800 embryos can be microinjected within 2 h, which provides enough zebrafish xenografts for the drug screening.

5-FU-based chemotherapy is currently the first-line treatment for GC. Several GC cell lines showed varied sensitivity to 5-FU treatment in vitro [39, 40] including SGC-7901 and AGS cells used in our research. Surprisingly, both SGC-7901 and AGS showed weak sensitivity to 5-FU treatment in the zebrafish xenograft model even treated with a soaking dosage of 4000 μM. The survival rate of embryos was 100% when treated by 500 μM of 5-FU by soaking. These results are quite different to those in Roel’s report, in which 5-FU at 500 μM administrated by soaking reduced tumor growth in zebrafish xenograft model, whereas the survival rate of embryos was 50% after 48 h soaking treatment [41]. The reported Log *P* value of 5-FU was −0.89 (https://www.drugbank.ca/drugs/DB00544, 20,170,504). Compound with Log *P* values less than 1 are typically not well-absorbed from the medium by zebrafish embryos [42, 43], which might cause the poor absorption of 5-FU by the zebrafish embryo by soaking. Therefore, we administered 5-FU to the embryos by microinjection. 5 and 50 μM of 5-FU were prepared in embryo medium, 10 nl of each drug medium was injected to the yolk sac of zebrafish embryo, and 5-FU at 6.5 and 65 ng/embryo caused obvious tumor cell growth regression.

Instead of embryo imaging followed by fluorescence density measurement, we quantified human cell growth through cell dissociation followed by fluorescent cell counting. Although tumor quantification via fluorescent imaging was used by many researchers [18, 23, 36, 44], we noticed that fluorescent-labeled cells were scattered in a three-dimensional fashion of the whole embryo after injection. Hence, the measurement of the photomicrographs based on the fluorescence density could not accurately reflect the cell number. Moreover, the red fluorescent density of a CM-DiI-labled cell tend to fade when the cell divided into two daughter cells, thus the fluorescent density would not be enhanced over the cell proliferation. Therefore, we dissociated the zebrafish embryos at indicated time point and manually counted the fluorescently-labeled cells [25]. This method showed much higher accuracy than quantification by imaging.

GC belongs to a type of cancer with high heterogeneity. The Cancer Genome Atlas (TCGA) has proposed a molecular classification dividing GC into four subtypes: 1) tumors positive for Epstein-Barr virus; 2) microsatellite unstable tumors; 3) genomically stable tumors; and 4) tumors with chromosomal instability [45]. The Asian Cancer Research Group (ACRG) also suggested four subtypes of GC and linked the distinct pattern of molecular alterations with disease progression and prognosis [46]. Based on these incredible progressions of the translational research of GC, personalized medicine is urgently needed. Fior’s group has recently reported the establishment of zPDX model for the chemosensitive profiling of colorectal therapy, and found that the correlation was 90% (4 out of 5) between patients and their zPDX in terms of chemosensitivity [24]. We believe that zPDX model of GC might also represent a promising platform to perform preclinical drug screening, and even for the real-time selection of chemotherapeutic drugs in the clinic, although more patient samples and longer follow-up of clinic responses should be accomplished. Moreover, in this study, since these patients has surgery and adjuvant 5-FU when they presumably had no evidence of gross disease, the context is somewhat different than the zebrafish studies where the 5-FU is given as primary therapy. But this does not devalue the zebrafish studies to determine tumor sensitivity to perhaps predict which patients would benefit from the addition of 5-FU.

## Conclusions

In conclusion, our research suggests the applicability of a new zPDX model as an innovative platform to the translational research including tumor-microenvironment interaction, biomarker discovery, and drug screening etc. Our future studies with a larger sample size will focus on investigating its potential utility in the therapeutic decision-making of GC.

## Additional files

Additional file 1: Supplementary Materials and Methods. (DOCX 21 kb)

Additional file 2: Figure S1. The survival rate of zebrafish xenografts at different temperatures. Zebrafish xenografts were incubated at 28 °C, 30 °C, 32 °C, and 34 °C respectively from 48 hpf to the indicated days post. Quantitative values are means ± SEM from 4 independent groups, with at least 10 embryos per group. Hpf: hours post fertilization. (JPEG 95 kb)

Additional file 3: Figure S2. The cell viability (presented as CCK-8 staining) of AGS and SGC-7901 under different culture temperature during 3 days incubation. Quantitative values are means ± SEM from 3 replicates at 24 h, 48 h, and 72 h after cell inoculation. (JPEG 76 kb)

Additional file 4: Figure S3. Fluorescent microscopy analysis of dissociated embryos. Xenografted embryos were dissociated and the resulting cell suspension were analyzed by fluorescent microscopy. The eight cells in the field of view that stain positive for CM-DiI colocalize with individual nuclei (white arrows) stained with DRAQ5 nuclear stain. (JPEG 50 kb)

Additional file 5: Figure S4. Cell viability assay of SGC-7901 and AGS to 5-FU treatment. SGC-7901 and AGS cell lines were treated with increasing concentrations of 5-FU (0–5000 μM) respectively for 72 h. Following 72 h treatment, cells were subjected to CCK-8 staining for viability. The percentage viability was plotted versus the drug dose. Quantitative values are means ± SEM from 3 replicates. (JPEG 114 kb)

Additional file 6: Figure S5. Toxicity curves for 5-FU, docetaxel, and apatinib. Zebrafish embryos at 72 hpf were treated with increasing concentrations of 5-FU (0–6500 ng/embryo), docetaxel (0–80 μM), and apatinib (0–50 μM) for 2 days. Following 2-day treatment, embryos were examined for viability and teratogenicity. The percentage viability and teratogenicity (for apatinib only) were plotted versus the drug dose. Quantitative values are means ± SEM from 3 replicates. *N* = 45 embryos at each dose level. (JPEG 125 kb)

## Abbreviations

5-FU: 5-Fluorouracil
ACRG: Asian Cancer Research Group
ATCC: American Tissue Culture Collection
CCK-8: Cell counting kit-8
CDX: Cell line-derived xenograft
Dpf: Days post fertilization
Dpi: Days post injection
Dpt: Days post treatment
EGFP: Enhanced green fluorescent protein
FBS: Fetal bovine serum
GC: Gastric cancer
Hpf: Hours post fertilization
IACUC: Institutional Animal Care and Use Committee
IARC: International Agency for Research on Cancer
IC_50_: half maximal inhibitory concentration
mPDX: mouse PDX
MTD: Maximum tolerated dose
NOD: Non-obese diabetic
PBS: phosphate buffer saline
PDX: Patient-derived xenograft
RPMI 1640: Roswell Park Memorial Institute basal medium 1640
SCID: Severe combined immunodeficiency
SIVs: Subintestinal vessels
TCGA: Cancer Genome Atlas
Tg: Transgenic
VRI: VEGFR tyrosine kinase inhibitor II
zPDX: zebrafish PDX
