# Supplementary material for: Patient-derived xenograft in zebrafish embryos: a new platform for translational research in gastric cancer
Source: J Exp Clin Cancer Res. 2017 Nov 15;36:160. doi: 10.1186/s13046-017-0631-0 (PMC5688753; doi:10.1186/s13046-017-0631-0)
Supplement: Supplementary file 1 — Supplementary Materials and Methods. (DOCX 21 kb) [file 13046_2017_631_MOESM1_ESM.docx]

**Supplementary Materials and Methods**

**Validation of the culture temperature for the zebrafish xenograft**

The zebrafish xenografts were maintained at 28°C, 30°C, 32°C, and 34°C respectively after cell injection from 48 hpf for the following 4 days. The survived embryos were counted every day. We found that the embryos survived best at ≤ 32°C (Fig. S1). Temperature equals to or higher than 34°C led to a significant decrease of the embryo survival rate (Fig. S1).

The AGS and SGC-7901 cell lines were inoculated in multi-well plate and cultured under 32°C and 37°C in their original culture medium for 3 days. Cell viability was measured by CCK-8 staining at each day. There were no significant differences in the viability of AGS and SGC-7901 cultured under 32°C compared with 37°C at day 1, 2 and 3 (Fig. S2). These results suggest that the cancer cells can tolerate and proliferate within a range of temperatures that include those suitable for the zebrafish host.

**Toxicity curve for maximum tolerated doses (MTD) determination**

Zebrafish embryos at 72 hpf were treated with increasing concentrations of 5-FU (0-6500 ng/embryo), docetaxel (0-80 μM), and apatinib (0-50 μM) for 2 days. Following 2 days treatment, embryos were examined for viability and teratogenicity. The percentage viability and teratogenicity (for apatinib only) were plotted versus the drug dose (Fig. S5). 5-FU and docetaxel showed decreased survival rate in a dose-dependent manner. Apatinib showed no lethal toxicity even at the highest concentration of 50 μM, but caused an obvious teratogenicity to embryos at concentrations higher than 0.5 μM. So, the maximum tolerated doses (MTD) for the following assay were 65 ng/embryo for 5-FU, 0.5 μM for apatinib, and 5 μM for docetaxel (Fig. S5)
