# Supplementary figures and images for: Patient-derived xenograft in zebrafish embryos: a new platform for translational research in gastric cancer
Source: J Exp Clin Cancer Res. 2017 Nov 15;36:160. doi: 10.1186/s13046-017-0631-0 (PMC5688753; doi:10.1186/s13046-017-0631-0)

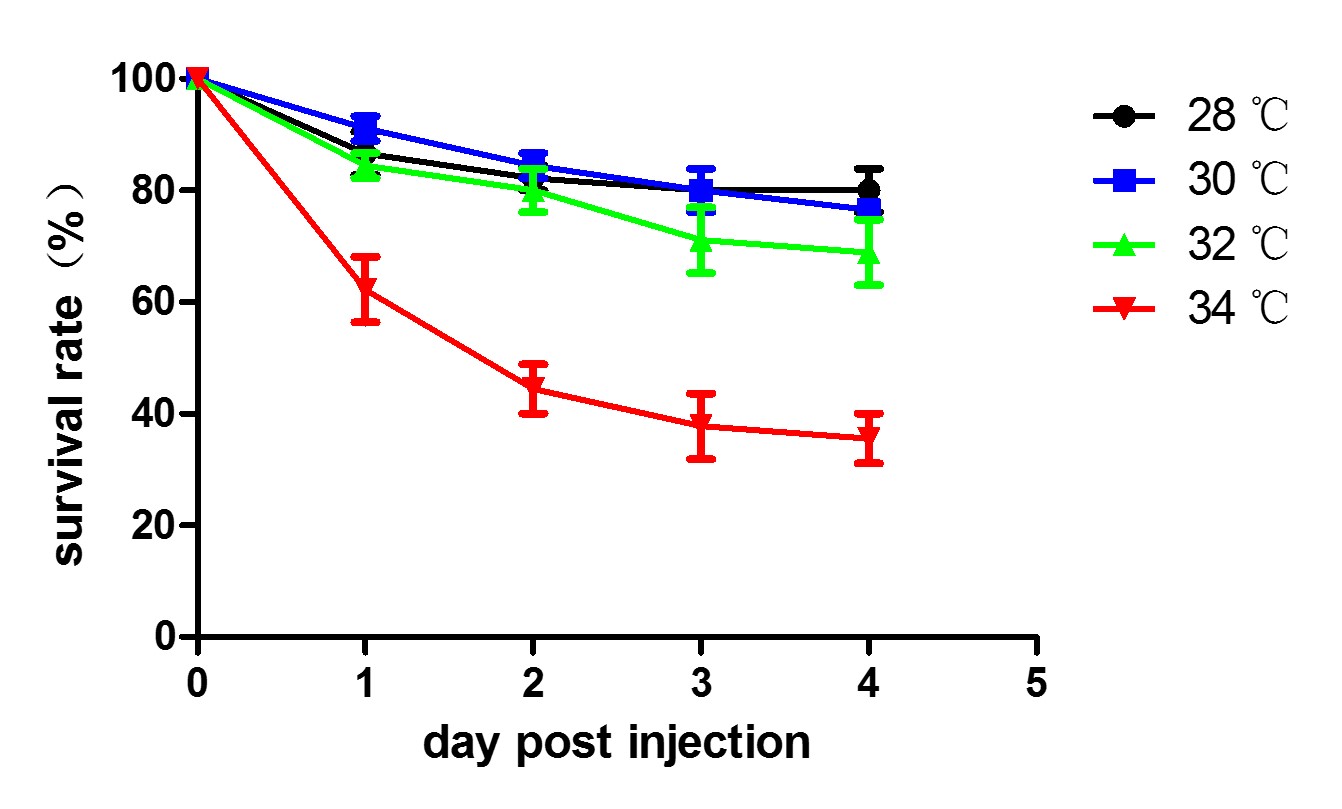

Supplement: Supplementary file 2 — The survival rate of zebrafish xenografts at different temperatures. Zebrafish xenografts were incubated at 28 °C, 30 °C, 32 °C, and 34 °C respectively from 48 hpf to the indicated days post. Quantitative values are means ± SEM from 4 independent groups, with at least 10 embryos per group. Hpf: hours post fertilization. (JPEG 95 kb) [file 13046_2017_631_MOESM2_ESM.jpg]

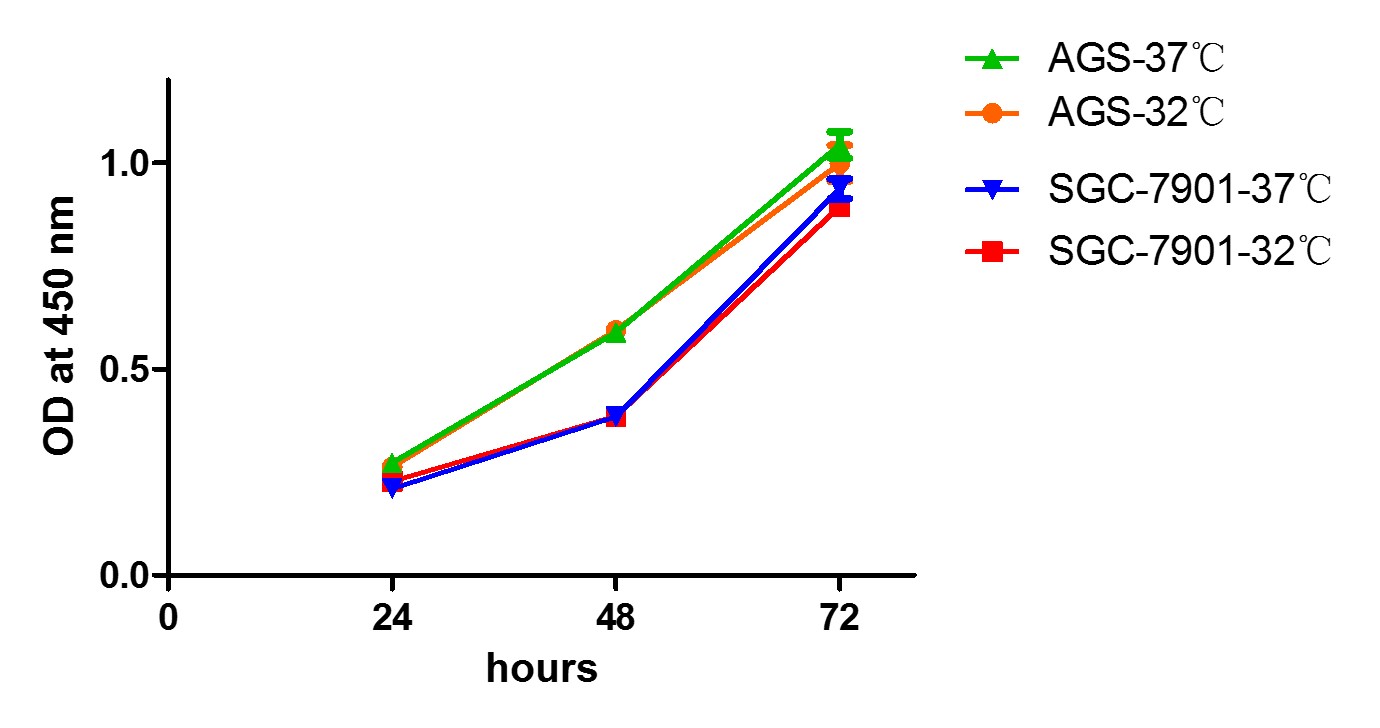

Supplement: Supplementary file 3 — The cell viability (presented as CCK-8 staining) of AGS and SGC-7901 under different culture temperature during 3 days incubation. Quantitative values are means ± SEM from 3 replicates at 24 h, 48 h, and 72 h after cell inoculation. (JPEG 76 kb) [file 13046_2017_631_MOESM3_ESM.jpg]

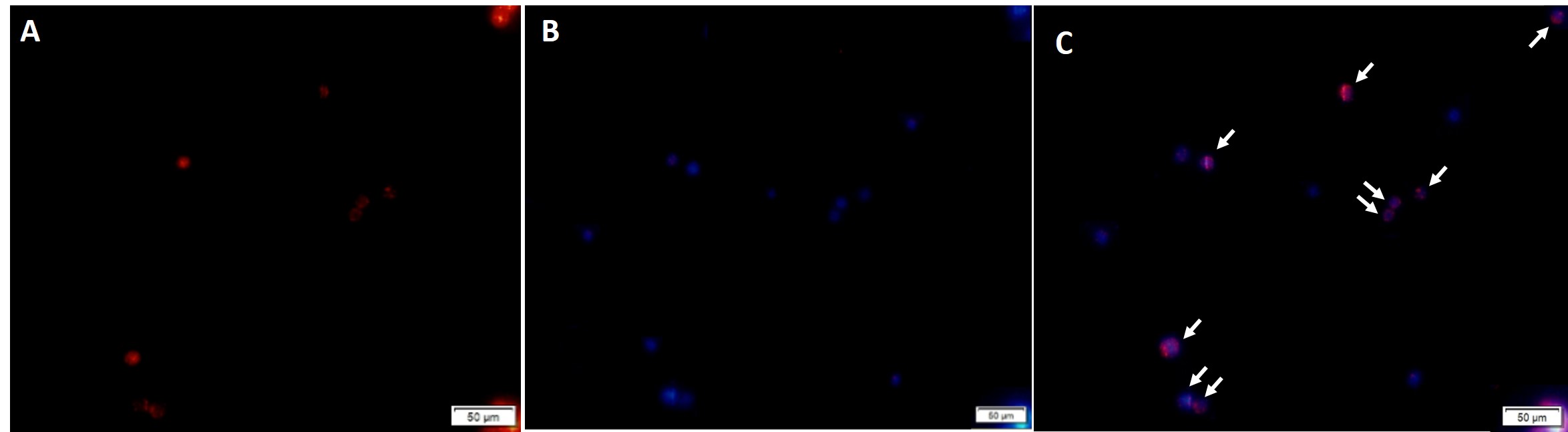

Supplement: Supplementary file 4 — Fluorescent microscopy analysis of dissociated embryos. Xenografted embryos were dissociated and the resulting cell suspension were analyzed by fluorescent microscopy. The eight cells in the field of view that stain positive for CM-DiI colocalize with individual nuclei (white arrows) stained with DRAQ5 nuclear stain. (JPEG 50 kb) [file 13046_2017_631_MOESM4_ESM.jpg]

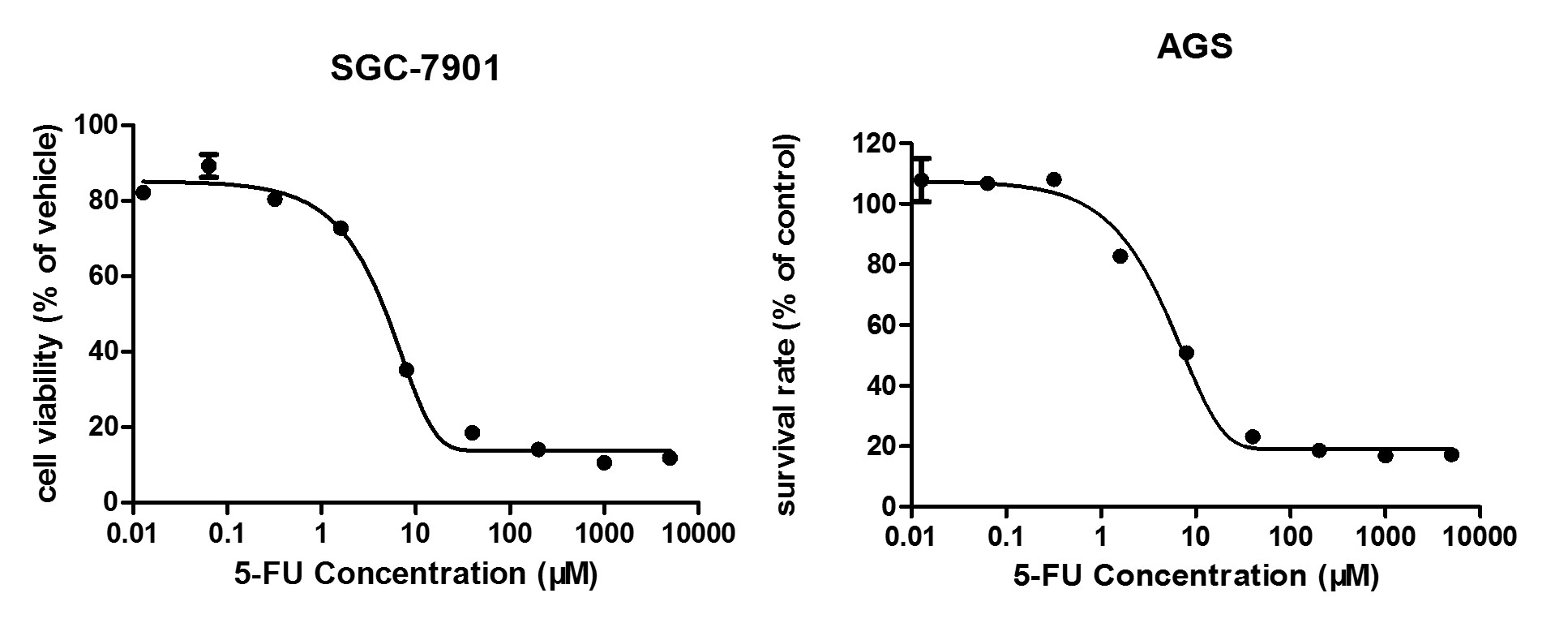

Supplement: Supplementary file 5 — Cell viability assay of SGC-7901 and AGS to 5-FU treatment. SGC-7901 and AGS cell lines were treated with increasing concentrations of 5-FU (0–5000 μM) respectively for 72 h. Following 72 h treatment, cells were subjected to CCK-8 staining for viability. The percentage viability was plotted versus the drug dose. Quantitative values are means ± SEM from 3 replicates. (JPEG 114 kb) [file 13046_2017_631_MOESM5_ESM.jpg]

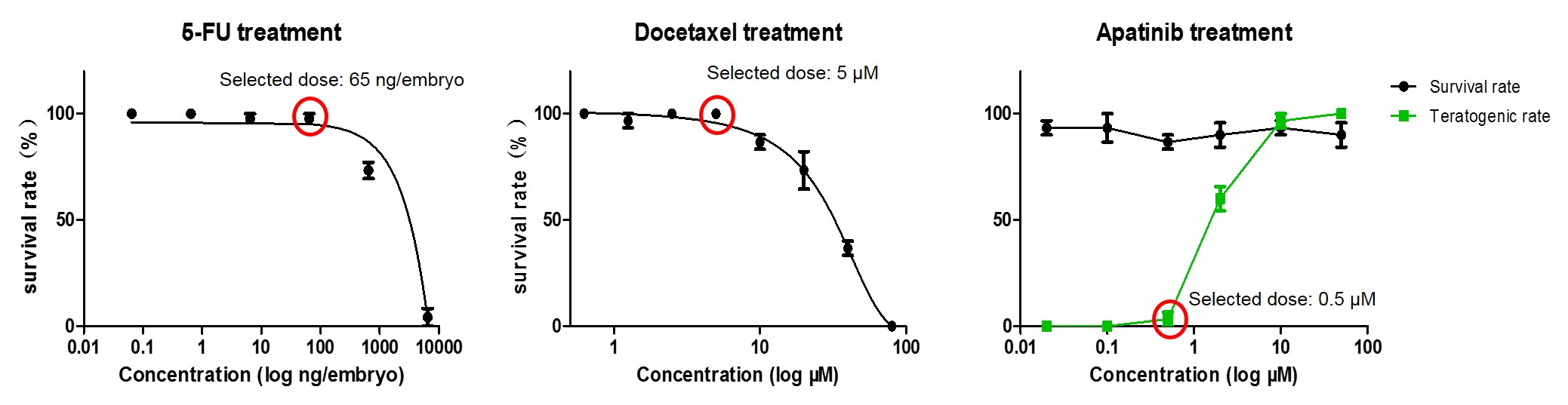

Supplement: Supplementary file 6 — Toxicity curves for 5-FU, docetaxel, and apatinib. Zebrafish embryos at 72 hpf were treated with increasing concentrations of 5-FU (0–6500 ng/embryo), docetaxel (0–80 μM), and apatinib (0–50 μM) for 2 days. Following 2-day treatment, embryos were examined for viability and teratogenicity. The percentage viability and teratogenicity (for apatinib only) were plotted versus the drug dose. Quantitative values are means ± SEM from 3 replicates. N = 45 embryos at each dose level. (JPEG 125 kb) [file 13046_2017_631_MOESM6_ESM.jpg]
